# Improving the Resistance Welding Quality of CF/PA6 Composite by Adjusting the Mesh Size of a Perforated Heating Element

**DOI:** 10.3390/polym18182275

**Published:** 2026-09-17

**Authors:** Shiyuan Wang, Zhanyi Geng, Yiwen Li, Chengyang Yi, Yuanduo Yang, Sansan Ao, Yang Li

**Affiliations:** 1 School of Materials Science and Engineering, Tianjin University, Tianjin 300354, China; w_sy07@tju.edu.cn (S.W.); gzy2023@tju.edu.cn (Z.G.); lyw1921@tju.edu.cn (Y.L.); ycy040820@tju.edu.cn (C.Y.); yyd1111@tju.edu.cn (Y.Y.); ao33@tju.edu.cn (S.A.); 2 Tianjin Key Laboratory of Advanced Joining Technology, Tianjin 300350, China

**Keywords:** resistance welding, thermoplastic composite, heating element, edge effect

## Abstract

During resistance welding of fiber-reinforced thermoplastic composites (FRTP), lower temperatures are observed at the edge of the welding area that is vertical to the direction of the current (longitudinal edge, L-edge). This paper proposes a perforated heating element (HE) with reduced edge mesh size to increase the temperature in the L-edge, thereby mitigating the edge effect during the resistance welding of FRTPs. By reducing the mesh size in the L-edges of the welding area, a temperature gradient can be created during the heating process, thereby increasing heat generation at the L-edges of the HE. Through experimental and numerical investigations, the influence of this HE on temperature evolution and joint performance during FRTP resistance welding was investigated. A resistance model was proposed to analyze the resistance and heat generation of regions with different mesh sizes. The results show that the tailored HE increased the temperature in the L-edge area. Furthermore, the reduced mesh size decreased the resistance of the HE in the welding area, mitigating the excessive temperature in the transverse edge (T-edge) (parallel to the current direction) of the welding area. The joints welded with the tailored HE achieved a lap-shear strength (LSS) of 26.6 ± 0.8 MPa, which is 31% higher than the LSS of joints welded with unmodified HE. The proposed resistance model could predict the changing trends of resistance and heat generation in regions with different mesh sizes at the qualitative level. Further work needs to be carried out to improve its quantitative prediction ability.

## 1. Introduction

Fiber-reinforced thermoplastic composites (FRTPs) are characterized by their light weight, high strength, weldability and recyclability [1,2,3,4]. In recent years, they have been widely used in the aerospace, rail transport and automotive industries [5]. As industrial production inevitably involves the manufacture of large-scale structural components or complex parts, the joining of FRTPs has become a crucial consideration.

Resistance welding of FRTPs has attracted widespread attention from researchers because of its simple equipment design, low cost, high controllability and relatively good stability [6,7,8,9]. It works by applying an electric current to the heating element (HE) at the welding interface. The electrical resistance generates Joule heat to melt the joint material, and external pressure is applied to achieve reliable interfacial bonding [10,11,12]. One challenge in resistance welding of FRTPs is the non-uniform temperature field at the welding interface caused by edge effects [13]. This edge effect presents itself in two ways: first, in the direction parallel to the welding current (the longitudinal direction of the welding interface), the temperature at the transverse edge is higher than that at the center of the welding area. This is because heat transfer from the HE exposed to the air is slower [14]. Second, vertically in the direction of the welding current (the transverse direction of the welding interface), the temperature at the longitudinal edge is lower than that at the center of the welding area. This is because the longitudinal edge is cooled by the colder layer of laminates closer to the welding interface, while the center area of the joint is heated by the HE, causing the temperature to rise [15]. The problem of the uneven temperature field mentioned above will worsen with the increase in welding area [16].

There are currently two main strategies to alleviate the uneven temperature field at the welding interface of resistance welding. The first strategy is to adjust the fixture structure, specifically by reducing the clamping distance (the distance between the electrode and the edge of the welding area) appropriately [17]. Shortening the clamping distance can reduce the part of HE exposed to air, improve the cooling effect of the copper electrode on the HE exposed to air, and thus avoid the adverse effects of high temperature rise of HE exposed to air on the welding interface [18]. However, Talbot et al. [19] indicated that the temperature field at the welding interface was highly sensitive to the change in the clamping distance. They found that when the clamping distance was 0.65 mm, the temperature at the transversal edges of the welding area was relatively close to that at the center of the welding area. When the clamping distance changed by only 0.2 mm, the temperature at the transversal edges was higher (increased by 0.2 mm) or lower (decreased by 0.2 mm) than the temperature at the center. Precise control of the clamping distance within a very narrow range reduced the process applicability of FRTP resistance welding. The second strategy involved optimizing the structure of the HE. Stavrov et al. [20] compared carbon fiber HEs and stainless steel mesh (SSM) HEs and pointed out that the SSM HEs were less sensitive to changes in process parameters and offered a wider process window. Dubé et al [21] sprayed a TiO_2_ coating on the surface of SSM HE to avoid the shunt effect caused by the contact between stainless steel and carbon fiber, thereby improving the uniformity of the welding temperature field and the stability of the welding process. Brassard et al. [22] proposed a polyetherimide (PEI)/multi-wall carbon nanotubes (MWCNT) nanocomposite HE to improve the temperature uniformity. However, it was still observed that the temperature in the transversal edge region at the welding area was higher than that in the center region.

Although the above two strategies have improved the temperature field uniformity of the welding interface to some extent, they have not touched upon the essence of causing temperature field non-uniformity. According to Refs. [14,15], the uneven temperature field in resistance welding of FRTPs is caused by the uneven heat dissipation of the entire welding structure, and current strategies have not had a significant impact on the heat dissipation process. To address this issue, we previously proposed a method to improve the uniformity of the temperature field, which uses a perforated SSM HE [23]. The perforated HE had a larger mesh size in the welding area and a smaller mesh size outside the welding area. Through such a setting, different regions of the HE have different heat generation, thereby offsetting the temperature field non-uniformity caused by different heat dissipation. However, our previous design only addressed the issue of high temperature at the transverse edge of the welding area. In this study, a novel perforated HE was proposed. The new design adjusted the mesh size along both the transverse and longitudinal edges of the HEs. This not only alleviated the problem of excessive temperatures in the transverse edge of the welding area but also increased the temperature in the longitudinal edge of the welding area, thereby reducing the temperature difference between the edge and the center of the welding area.

## 2. Materials and Methods

### 2.1. Materials

**Figure 1:**
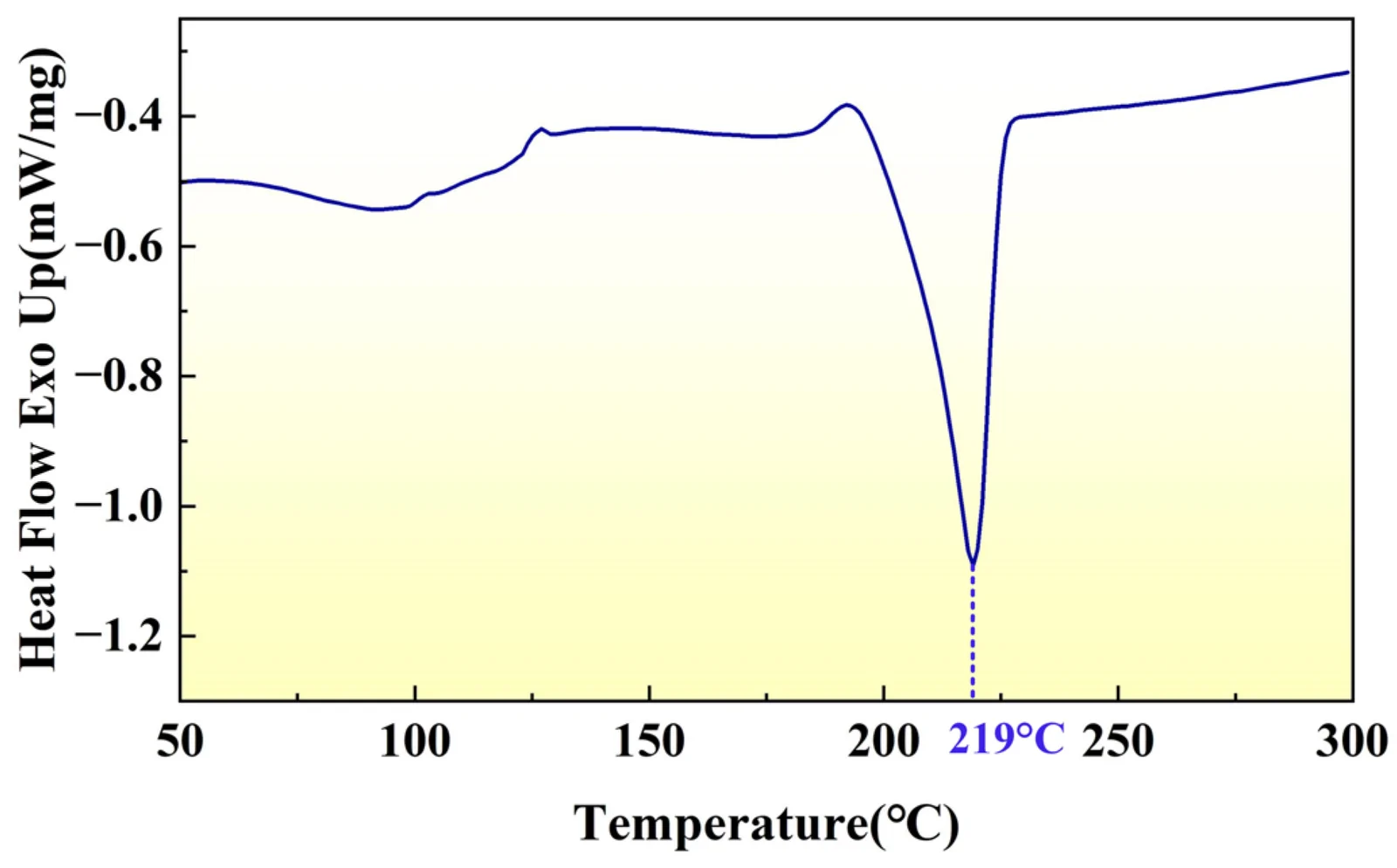
The DSC test curve of the PA6 resin used in this research.

Carbon-fiber-reinforced polyamide 6 (CF/PA6), which consisted of orthogonally woven T300-3K carbon fiber and Jin Young B18L injection-molding grade PA6 resin, was adopted as the experimental material (supplied by Jinyoung (Xiamen) Advanced Materials Technology Co., Ltd., Xiamen, China). The carbon fiber mass fraction was 60 wt%. The dimensions of the CF/PA6 laminates were 100 mm × 25 mm × 2 mm. Pure PA6 resin films were used as the insulating layers. Each film had dimensions of 25 mm × 20 mm × 0.2 mm. Prior to the experiment, the CF/PA6 laminates and the PA6 films were dried at 90 °C for 4 h and 2 h to remove the residual moisture, respectively. The melting point of the PA6 resin was determined to be 219 °C using differential scanning calorimetry (DSC; DSC 214, NETZSCH, Selb, Germany) (Figure 1).

### 2.2. Welding Diagram and Experimental Method

**Figure 2:**
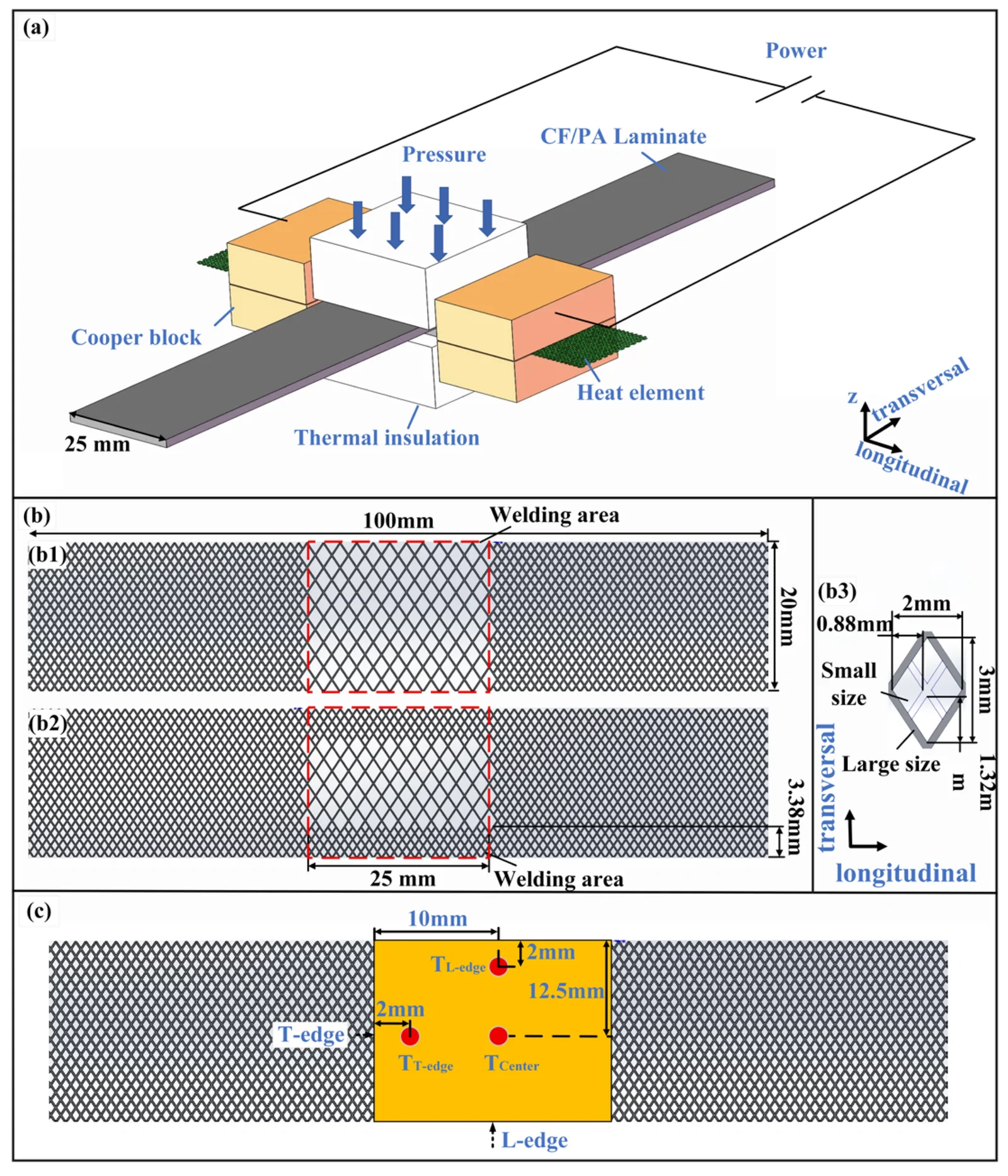
Schematic diagram of the (**a**) welding set-up, (**b**) dimensions of the (**b1**) Type A HE, (**b2**) Type B HE, and (**b3**) large and small meshes, (**c**) thermocouple temperature measurement positions.

Figure 2a shows the welding set-up. An SSM HE was placed between two laminates. Two PA6 films were placed between the HE and the CF/PA6 laminates to serve as the insulation. Both ends of the HE were clamped by copper blocks (copper electrodes), which were connected to the positive and negative poles of a power supply (Aikedes IT-9000, ITECH Electronic Co., Ltd., Nanjing, China). The clamping distance, defined as the distance from the copper electrode to the edge of the welding area, was 3.5 mm. A torque wrench was used to control the clamping pressure applied to the HE by the copper blocks to ensure tight contact between the copper blocks and the HE. The clamping pressure was 0.5 MPa. Thermal insulation ceramics were placed above and below the CF/PA6 laminates to achieve insulation and reduce heat loss during welding. The welding pressure was provided by a pneumatic pressurization device and applied to the upper insulating ceramic.

Two types of HE were used in this study, as shown in Figure 2(b1,b2). They were manufactured using an etching process and supplied by Dongguan Gongxiang Hardware Electronic Co., Ltd., Dongguan, China. The HE shown in Figure 2(b1) is named Type A, which is characterized by the use of large mesh throughout the welding area and small mesh outside the welding area. The HE shown in Figure 2(b2) is named Type B, which is characterized by the use of small mesh at the edges of the welding areas on both the longitudinal and transversal sides, with the small-mesh area accounting for 33.8% of the welding area. The dimensions of the large and small meshes are shown in Figure 2(b3). The large mesh was 2 mm × 3 mm and the small mesh was 0.88 mm × 1.32 mm. The overall dimensions of the HEs were 100 mm × 20 mm × 0.2 mm. The area enclosed by the red outline denotes the welding area (20 mm × 25 mm). The longitudinal edge (L-edge) and transverse edge (T-edge) of the welding area are marked in Figure 2c.

In this study, welding was carried out in constant-current mode. The optimal welding parameters for the Type A HE were determined through preliminary experiments. The welding current was 35 A, welding time was 55 s, holding time was 55 s and welding pressure was 0.4 MPa. The same welding parameters were applied to the Type B HE for comparison. Six specimens were welded for each set of parameters; of these, five were used to measure the lap shear strength (LSS), and one was used for macrostructural and microstructural observation. A fracture failure analysis was carried out on the specimen exhibiting the highest LSS. The LSS was obtained by dividing the maximum lap shear force by the welding area (20 mm × 25 mm).

### 2.3. Morphology Characterization and Mechanical Testing

**Figure 3:**
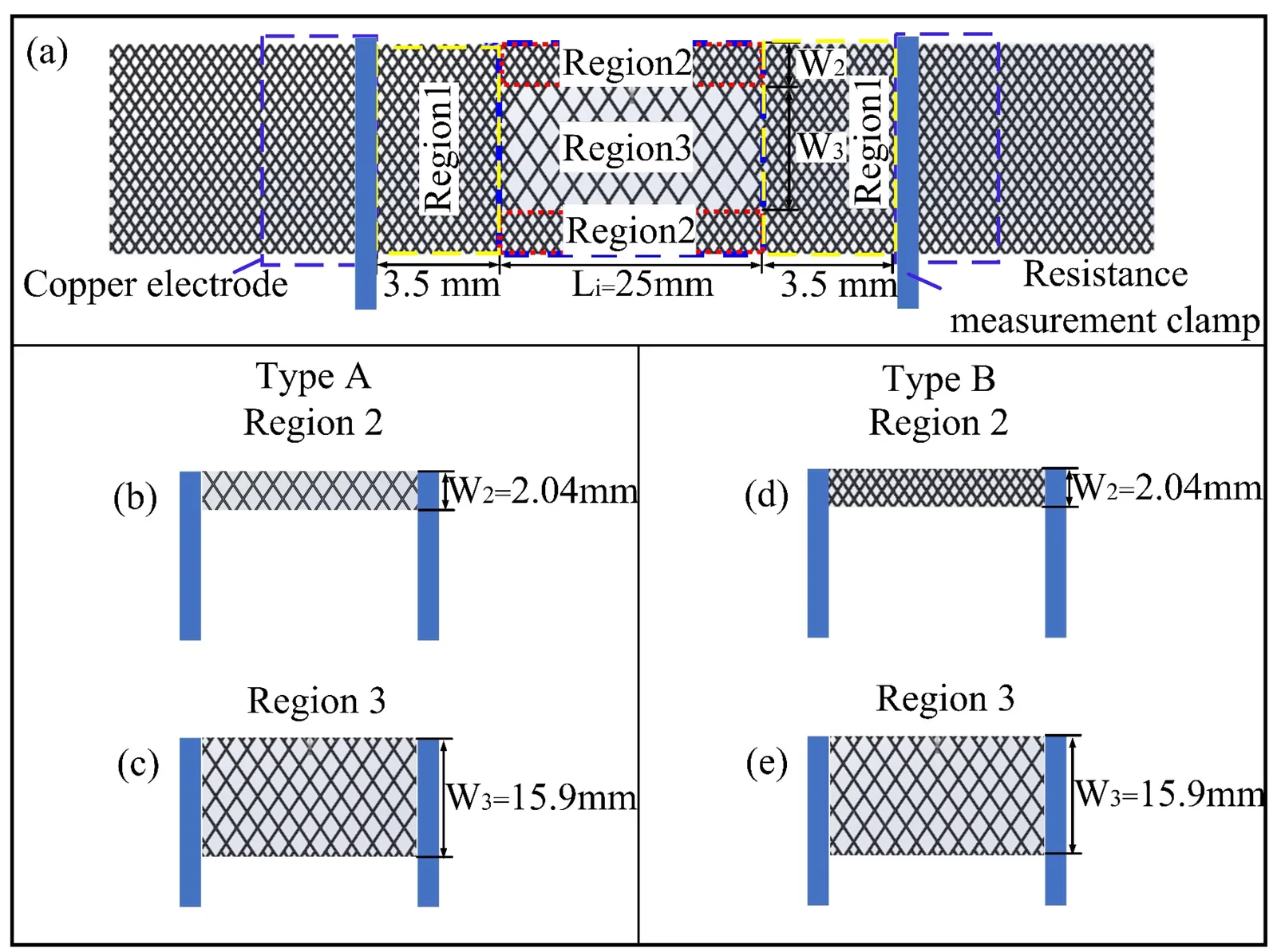
Schematic diagram for the resistance measurement: (**a**) total resistance measurement; (**b**) region 2 of the Type A HE; (**c**) region 3 of the Type A HE; (**d**) region 2 of the Type B HE; (**e**) region 3 of the Type B HE.

In this study, the lap shear test was carried out using a universal tensile testing machine (ETM105D) to obtain the LSS of welded joints. An ultra-depth-of-field microscope (Zeiss Smart zoom 5) and an optical microscope (Zeiss Axio Vert. A1, Zeiss, Oberkochen, Germany) were used to observe the macroscopic morphology and microstructure characteristics of the cross-section of welded joints. The actual resistance of the Type A and Type B HEs was measured using an HPS2511 DC resistance tester provided by Changzhou HELPASS Electronics Technology Co., Ltd., Changzhou, China, as shown in Figure 3a (only Type B HE is plotted; the measuring position for Type A HE was the same). The distance between the testing clamp and the edge of the welding area was 3.5 mm, which is consistent with the clamping distance. Both the Type A and Type B HEs can be divided into five regions, as shown in Figure 3a. The resistances of each region for each type of HE were also measured, as shown in Figure 3b–d.

Since the welding interface is closed, the temperature at the welding interface was measured by thermocouples, which is the mainstream method for measuring the interface temperature in the research on FRTP resistance welding [24,25,26]. K-type thermocouples with a diameter of 0.125 mm (OMEGA CHAL-005, Omega, Norwalk, CT, USA) were adopted to measure the temperature at the center (T_Center_), the longitudinal edge (T_L-edge_) and the transverse edge (T_T-edge_) of the welding area. In the z-direction, the thermocouples were located between the upper CF/PA6 laminate and the PA6 film; in the x-y direction, the temperature measurement location is shown in Figure 2c. The sample frequency was 10 Hz and the response time was 10 ms, which is much shorter than the welding time, rendering the thermal lag effect negligible. For temperatures above 0 °C, this K-type thermocouple has a measurement error of ±2.2 °C or ±0.75% of the measured temperature (provided by the OMEGA official website). Scanning electron microscopy (Thermo Fisher Scientific Quattro S, Thermo Fisher, Waltham, MA, USA) was used to observe and analyze the fracture surface after the lap shear test.

## 3. Results and Discussion

### 3.1. Joint Mechanical Performance

**Figure 4:**
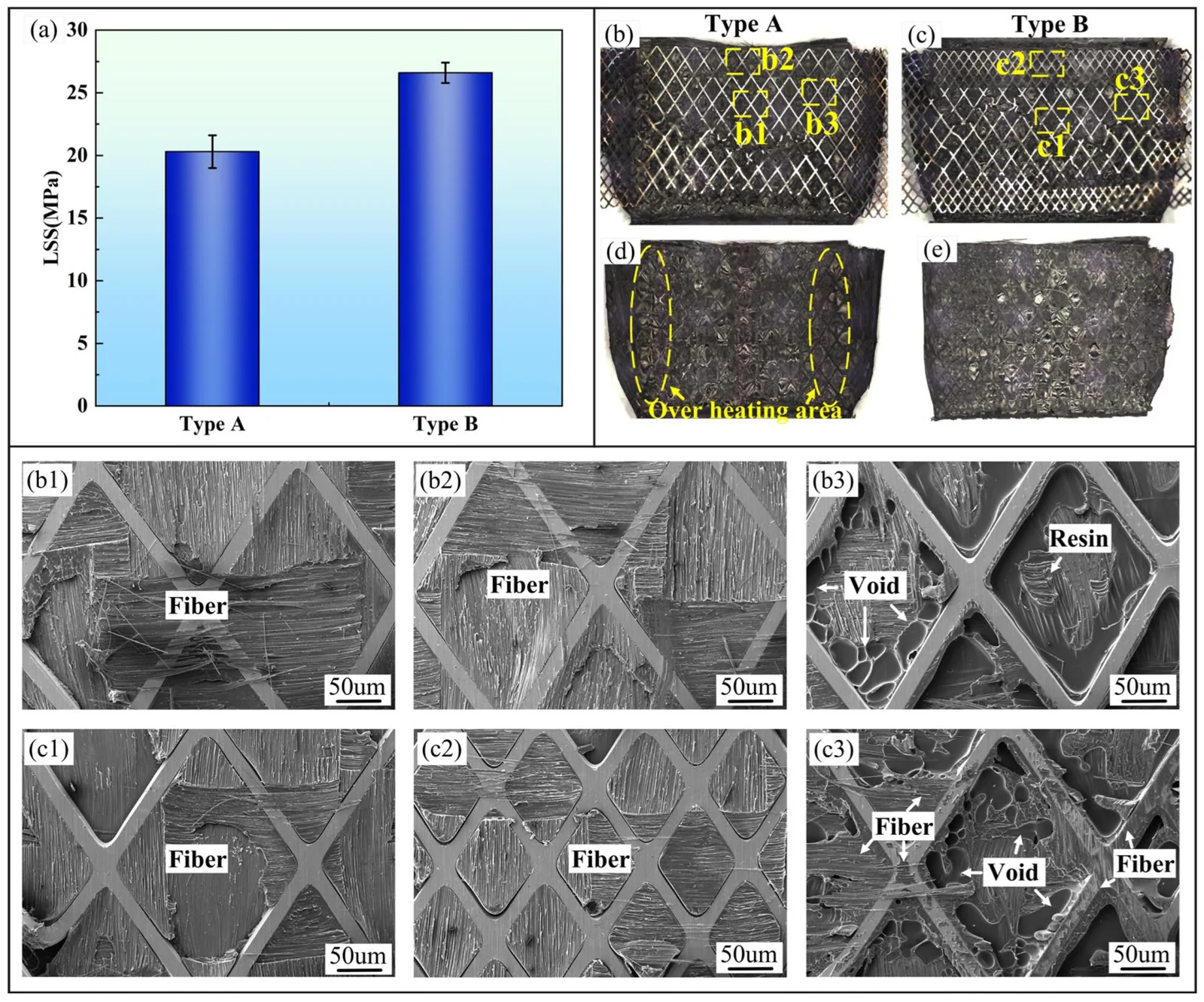
Mechanical performance of welded joints: (**a**) comparison of the LSS; (**b**,**d**) macroscopic morphology of the fracture surfaces of Type A HE welded joints; (**c**,**e**) macroscopic morphology of the fracture surfaces of Type B HE welded joints; (**b1**–**b3**) the enlarged fracture morphology marked in (**b**); (**c1**–**c3**) the enlarged fracture morphology marked in (**c**).

Figure 4a compares the LSS of joints made with the Type A and Type B HEs. The average LSS of joints made with the Type A HE was 20.3 ± 1.3 MPa, and that of joints made with the Type B HE was 26.6 ± 0.8 MPa. The LSS of the welded joints using the Type B HE is 31% higher than that of the welded joints using the Type A HE. It should be noted that this improvement reflects the joint performance under identical welding parameters, rather than the performance under conditions specifically optimized for each HE design.

Select one joint from the welded joints manufactured by the Type A and Type B HEs for the fracture morphology analysis. The selected joint made with the Type A HE had an LSS of 19.2 MPa (Figure 4b,d), and the selected joint made with the Type B HE had an LSS of 26.4 Ma (Figure 4c,e), both of which are close to their respective average values. Figure 4b,c show that tearing occurred in both HEs, indicating that adequate bonding was achieved in the welded joints. Figure 4d reveals a distinct overheating phenomenon in the transverse edge of the welding area of the Type A welded joint.

Microscopic analysis of the fracture surfaces of joints made with the two types of HE under identical welding parameters revealed that the mesh openings in both the center welding area and the edge of the welding area of the Type A and Type B HEs were fully filled with resin, as shown in Figure 4(b1–c3). The presence of detached fibers on the resin surface indicates that an effective bond was formed and that no obvious defects were present (Figure 4(b1,b2,c1,c2)). The failure mode in the center and transverse edge of the welding areas for Type A and B HEs was interlaminar failure [14]. However, in the transverse edge area of the Type A HE joint, a large number of voids and unfilled regions were observed within the mesh (Figure 4(b3)), and fiber impression marks were present on the surface of some of the resin filling, indicating that the bond in this area was insufficient. In contrast, the transverse edge area of the Type B HE joint exhibited a relatively high degree of resin filling within the mesh, and the presence of peeled fibers on the surfaces of both the resin and the metal mesh indicated a good bond in this area (Figure 4(c3)).

### 3.2. Joint Cross-Sectional Morphology

**Figure 5:**
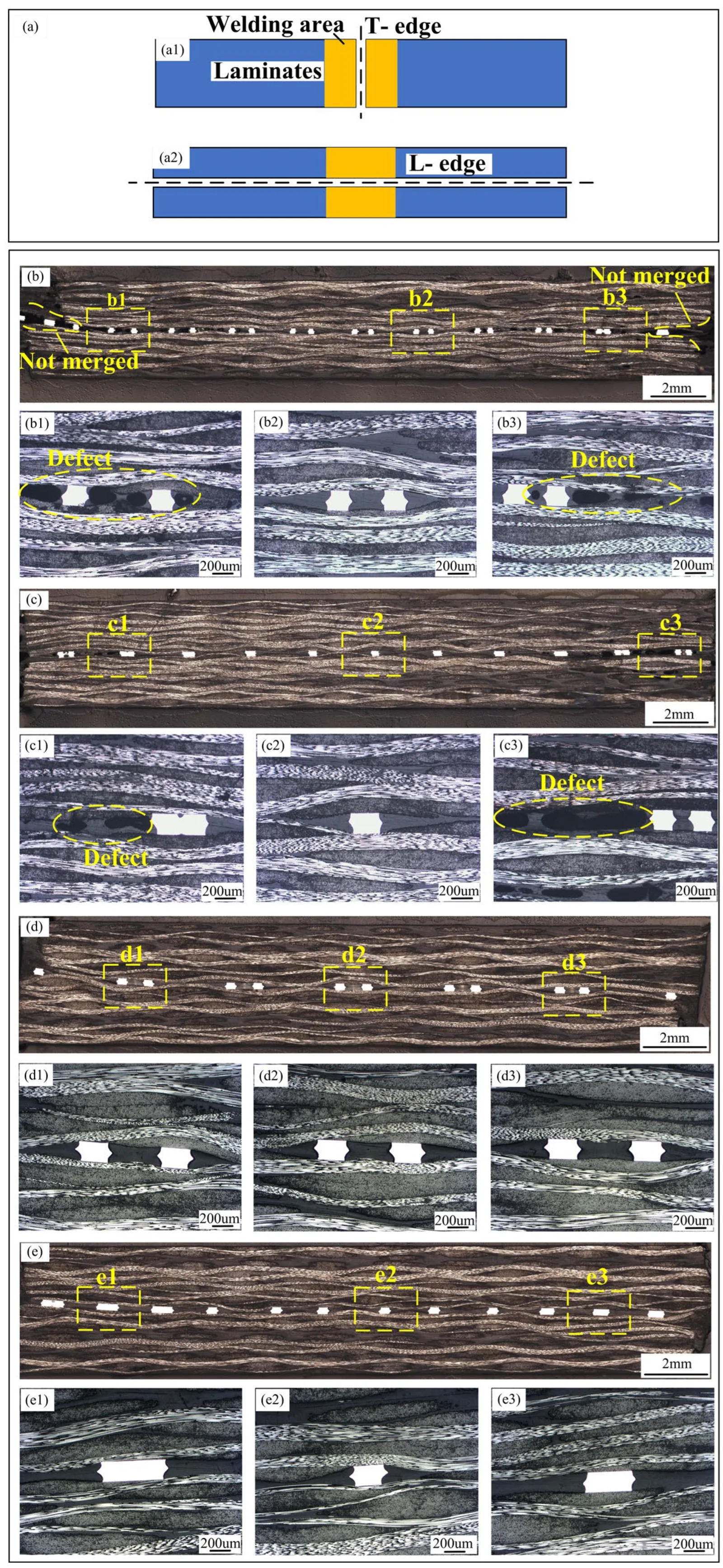
Longitudinal and transversal cross-sectional morphologies of the joints made with the Type A and Type B HEs: (**a**) cutting along the (**a1**) longitudinal direction and (**a2**) transversal direction; (**b**) longitudinal cross-section of the joint made with the Type A HE; (**c**) longitudinal cross-section of the joint made with the Type B; (**d**) transversal cross-section of the joint made with the Type A HE; (**e**) transversal cross-section of the joint made with the Type B HE. (**b1**–**b3**), (**c1**–**c3**), (**d1**–**d3**), and (**e1**–**e3**) show the enlarged images marked in (**b**), (**c**), (**d**) and (**e**), respectively.

Figure 5 shows the macroscopic and microscopic cross-sectional morphologies of joints made with the Type A and Type B HEs. Figure 5a shows the cutting direction. As can be seen from Figure 5b,c, distinct welded defects are present at the T-edges of the welded interfaces for both types of HEs. As can be seen from Figure 5d,e, no obvious defects can be observed at the edges or in the center of the welding area for either type of HE in the longitudinal direction.

**Figure 6:**
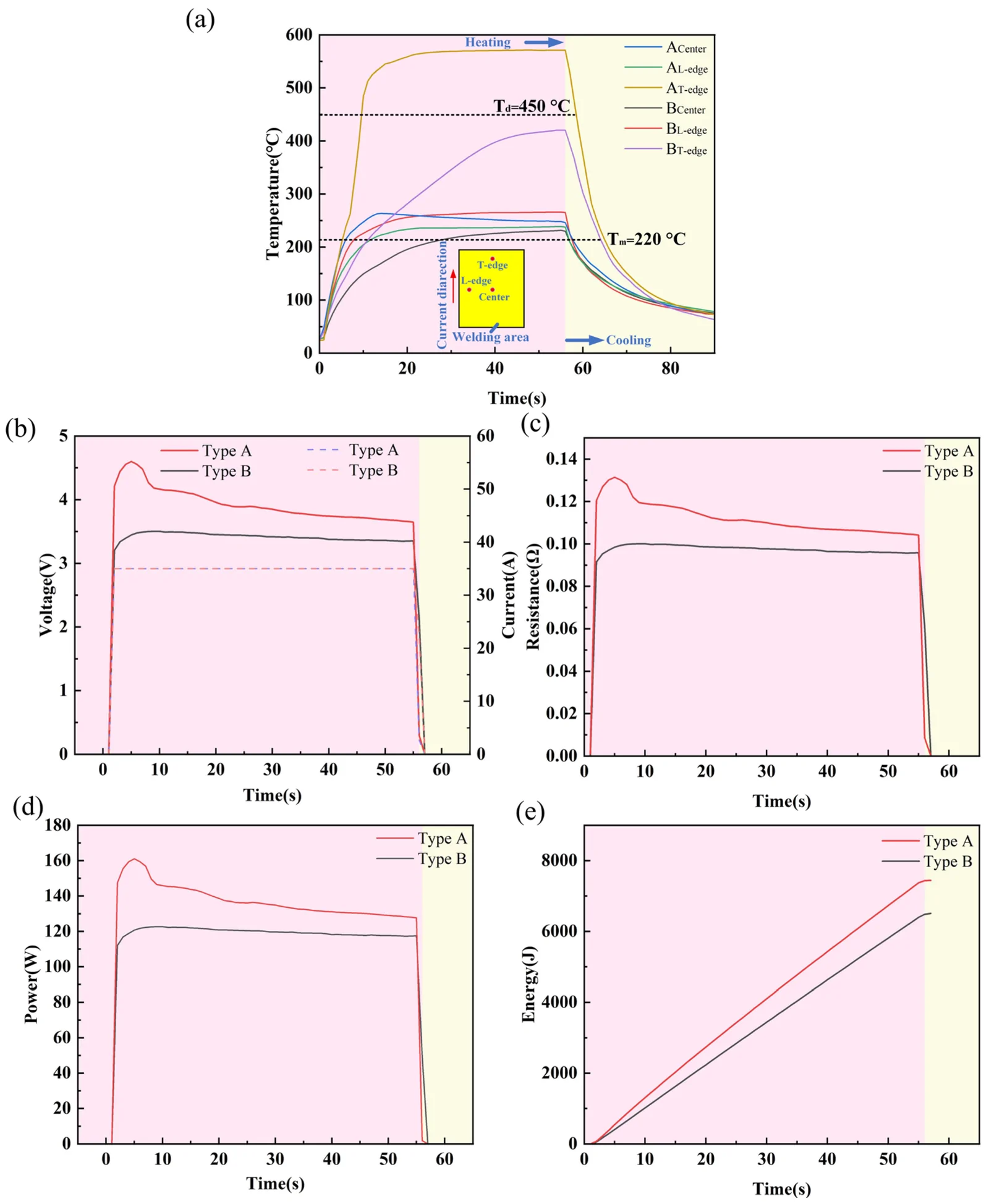
Welding temperature and parameter curves for the Type A and B HEs: (**a**) temperature curve on the welding interface; (**b**) voltage and current curves; (**c**) resistance curves; (**d**) power curves; (**e**) energy curves.

The temperature measurement results are used to analyze the cross-sectional morphologies. Figure 6a shows the measured temperature curves at the center, the longitudinal edge and the transverse edge of the welding area. For the Type A joint, the maximum temperature at the T-edge reached 571 °C, exceeding the thermal decomposition temperature of PA6 (450 °C [26,27]). Void defects at the T-edge may be caused by the thermal decomposition of the resin due to high temperature [6], as shown in Figure 5b. The maximum temperature at the center of the welding area for the Type A joint was 262 °C, higher than the maximum temperature at its L-edge (238 °C). This phenomenon is consistent with the existing literature reports, and the reasons for this have already been explained in the Introduction section. For the Type B joint, the maximum temperature at the T-edge was 420 °C, which did not exceed the thermal decomposition point. However, voids were still observed in the cross-section of the joint (Figure 5c), possibly due to insufficient resin flow or thermal decomposition that may occur in the actual resin system at temperatures below the thermal decomposition temperature [6]. The temperature at the center of the welding area for the Type B joint was 231 °C, which was lower than the maximum temperature at its L-edge (265 °C). This indicates that the problem of insufficient temperature at the L-edge in traditional resistance welding can be resolved by reducing the mesh size close to the L-edge.

To better understand the effects of the two types of HE on the welding process, we analyzed the signal curves during welding, including voltage, current, resistance, power, and energy, as shown in Figure 6b–d. The voltage, current, and power curves were provided by the welding power source; the resistance curve was obtained by dividing voltage by current; and the energy curve was derived by integrating power over time.

As shown in Figure 6b, the voltage curve for the Type A HE rose initially and then declined, while the voltage curve for the Type B HE rose initially and then leveled off. Since a constant-current mode was used in this study, the shape of the resistance (U/I) curve and the power curve (U × I) is essentially identical to that of the voltage curve, as shown in Figure 6c and Figure 6d, respectively. As can be seen in Figure 6d, the energy input for the Type A HE was higher than that for the Type B HE, and the energy curve showed an upward trend until the end of the welding.

For the Type A HE, the resistance curve showed a gradual decline, while the resistance for the Type B HE remained nearly constant. Generally, the resistivity of stainless steel increases with rising temperature. However, in this study, the resistance curve did not show an increase over time. This indicates that during the welding process, as the insulating film melted, the HE came into contact with the carbon fiber matrix, causing current diversion [17]. This phenomenon leads to a decrease in resistance. Previous studies have shown that current diversion during welding can cause the resistance to plateau or even decrease, with changes in the resistance curve being influenced by both temperature and current diversion [17]. At the same welding parameters, the resistance curve of the Type B HE exhibited lower fluctuations, indicating that current leakage is less severe compared with the Type A HE. Additionally, the resistance of the Type A HE remained consistently higher than that of the Type B HE. This phenomenon is consistent with the premise of this study: the Type B HE contains more metal wires (finer mesh) than the Type A HE, and therefore, its resistance should theoretically be lower than that of the Type A HE (this is also consistent with the theoretical calculation presented later in this paper).

From the energy curve, the Type A HE generated more heat due to its higher resistance, which is consistent with its higher temperature during the welding process (Figure 6a). Although the Type B HE generated less heat, its joint strength was higher than that of the Type A HE. This indicates that although Type A generated more heat, its non-uniform interface temperature distribution actually led to a decline in weld quality. For the Type B HE, although the welding parameters used in this study may not necessarily be optimal, it reduced the temperature difference between the edge and the center of the welding area, thereby enabling the achievement of higher-strength joints at lower energy levels—a factor of great importance for engineering application. These findings confirm the validity of the HE optimization strategy proposed in this study; we will conduct more systematic and in-depth research on mesh structure optimization and process optimization in the future.

### 3.3. Simulation of Heat Generation in the Tailored Mesh HE

**Figure 7:**
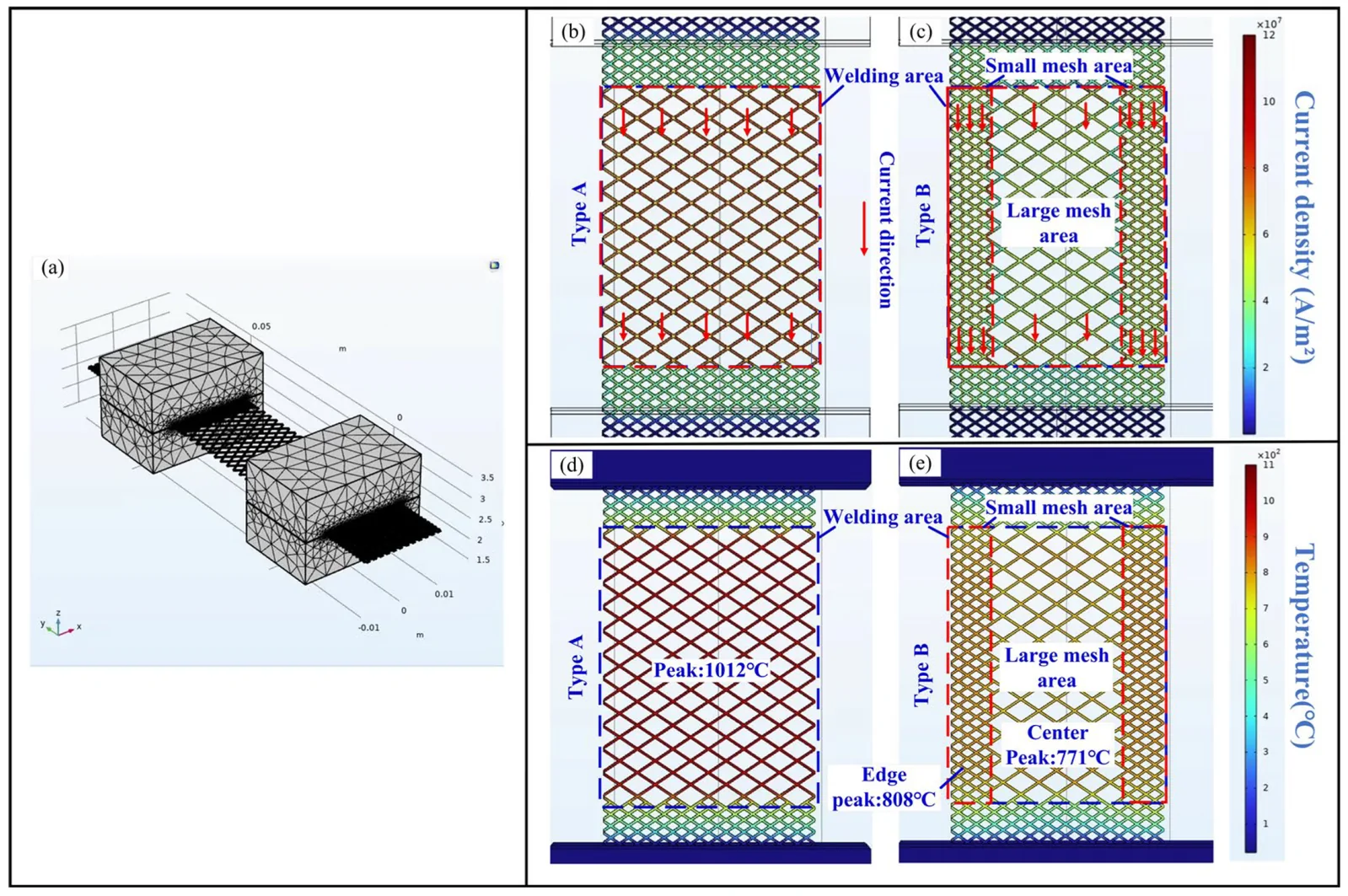
Temperature Simulation results for the Type A and Type B HEs: (**a**) finite element model and meshing; (**b**) current density distribution in the Type A HE; (**c**) current density distribution in the Type B HE; (**d**) temperature distribution in the Type A HE; (**e**) temperature distribution in the Type B HE.

This study investigated the effect of varying the mesh size of the HE on heat generation through transient heat transfer simulation. COMSOL software (COMSOL Multiphysics^®^ 6.2) was used to conduct the simulation, and the model comprises copper block electrodes and a 304 perforated SSM HE, as shown in Figure 7a. The material properties of the 304 stainless steel are shown in Table 1. The contact area between the copper electrodes and the HE is a rectangle formed by the width of the heating element and the width of the copper block. A constant current of 35 A was applied to the bottom surface of one copper electrode block, while the bottom surface of the other copper electrode block was grounded. The electrical contact between the copper block and the HE was modeled using the “Electrical Contact” setting; only the pressure and the materials’ properties were allowed to be input, and the contact resistance was calculated by the built-in algorithm of COMSOL.

Several assumptions were made to simplify the simulation process.

(1) The transient heat transfer simulation was only adopted to analyze the heat generation and current density distribution of the heating element, instead of simulating the actual welding process. The CF/PA6 laminates and the PA6 films were not involved in the FE model, and therefore, the simulation is not used for predicting or verifying the temperature at the welding interface during the actual welding process.

(2) To reduce the number of coupled physical fields and improve computational efficiency, the pressure between the copper electrodes on both sides of the heating element was defined via the electrical contact module in the model.

(3) The copper electrodes on both sides hardly generated heat and exhibited fast heat conduction. Therefore, the material properties of the copper electrodes were considered as temperature-independent. The electrical conductivity of the copper electrodes was set as 5.8 × 10^7^ S/m.

Figure 7b,c shows that the current density in the welding area of the Type A HE is uniformly distributed, while in the Type B HE, the current density in the large mesh area is lower than that in the small mesh area.

To analyze the heat distribution of the HE from the perspective of current density distribution, the heat generated per time per unit volume of the HE follows Joule’s law (Equation (1)):

(1) $$q=J^{2}/\sigma$$

where *q* is the volumetric heat generation power per unit volume, σ is the electrical conductivity of the conductor, and *J* is the current density at a certain point of the conductor.

Figure 7d,e shows that the peak temperature of Type A HEs is 1012 °C, while that of Type B HEs is 808 °C. The temperature distribution in the welding area of the Type A HE is uniform, while in the Type B HE, the temperature in the large mesh area is lower than that in the small mesh area, with a difference in peak temperature of 37 °C. This indicates that refining the mesh size in the longitudinal edge of the welding area helps to increase heat generation in that area and reduces the electrical resistance within the welding area, thereby reducing heat generation within the welding area.

### 3.4. Design Method for Perforated Mesh HE Based on Equivalent Resistivity

**Figure 8:**
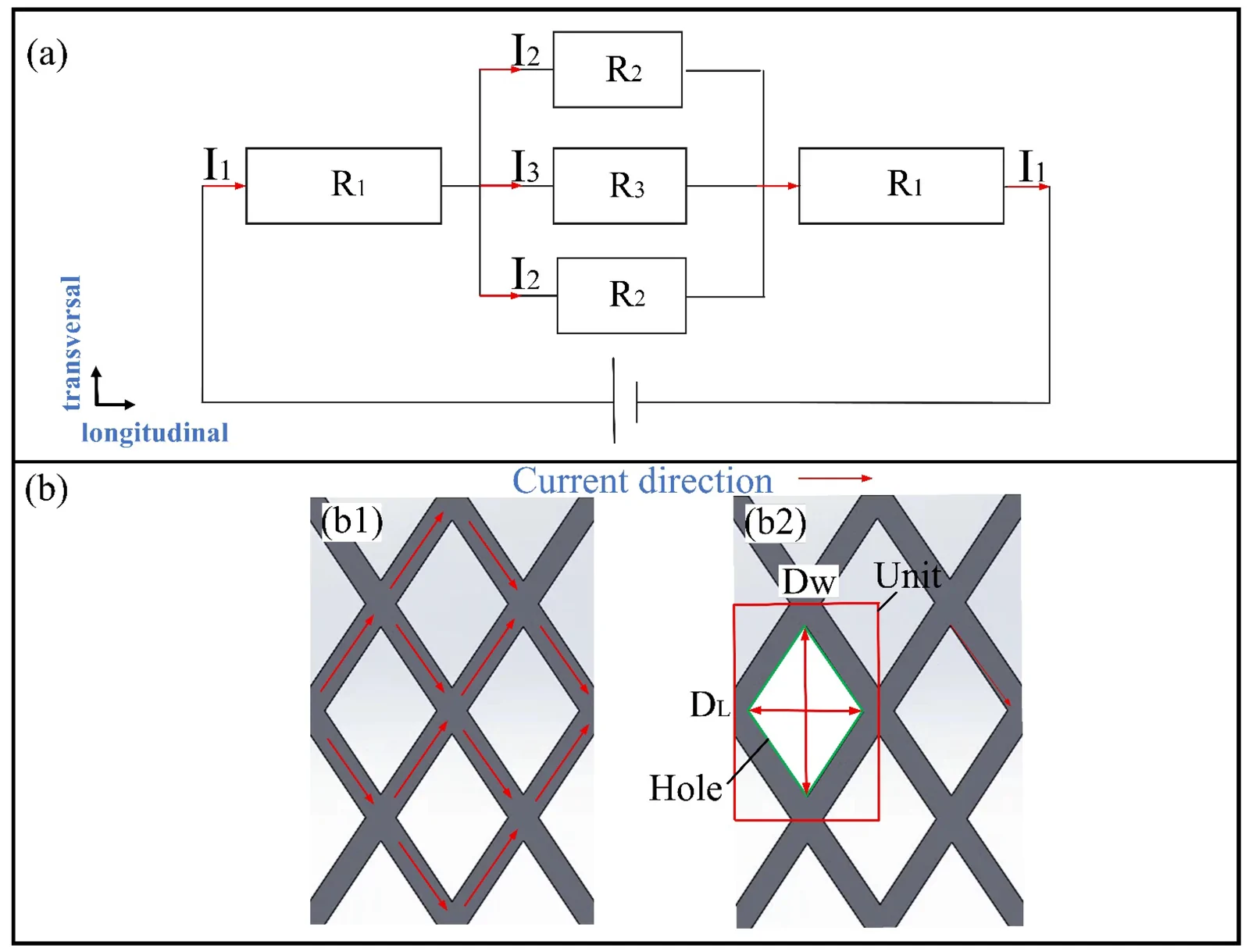
Schematic diagram of region equivalent resistance: (**a**) equivalent circuit diagram; (**b**) analysis of mesh resistance; (**b1**) current path; (**b2**) dimensions of one repeating unit.

This paper establishes a resistance model for the design of the perforated mesh HEs based on equivalent resistivity. Both the Type A and Type B HEs are divided into five regions, as shown in Figure 3a. Because of the symmetry of the HE, the resistances of the five regions can be denoted as *R*_1_, *R*_2_, and *R*_3_, respectively. When a welding current is applied to the HE, the entire system can be modeled as an equivalent circuit, as shown in Figure 8a. By changing the mesh size, the resistance values of *R*_1_, *R*_2_, and *R*_3_ can be adjusted to vary the heat generation in different regions, thereby mitigating the temperature non-uniformity caused by the edge effect.

Assuming the total welding current is *I*_1_, and the welding current passing through region 2 and region 3 is *I*_2_ and *I*_3_, respectively. Then, the heat generation in region 2 (*Q*_2_) and region 3 (*Q*_3_) can be calculated by solving the following equation group:

(2) $$\left\{\begin{array}{l} 2I_{2}+I_{3}=I_{1}\\ I_{2}R_{2}=I_{3}R_{3}\\ Q_{i}={I_{i}}^{2}R_{i}t\;\;\;\;(i=2,3) \end{array}\right.$$

where *t* is the welding time; *Q* is the total heat produced. By solving Equation (1), we can obtain

(3) $$Q_{2}={I_{2}}^{2}R_{2}t=\frac{I^{2}R_{3}^{2}R_{2}t}{{\left(2R_{3}+R_{2}\right)}^{2}}$$

(4) $$Q_{3}={I_{3}}^{2}R_{3}t=\frac{I^{2}R_{2}^{2}R_{3}t}{{\left(2R_{3}+R_{2}\right)}^{2}}$$

The resistance of each region is calculated based on an equivalent resistivity method. For an HE that is designed with a diamond-shaped hole structure, when the longitudinal current encounters a hole, it flows along the metal wires that surround the hole, causing current diversion and forming a parallel circuit, as shown in Figure 8(b1). For an SSM composed of diamond-shaped holes, a rectangle formed by the centerlines along the long and short axes of two adjacent diamond-shaped holes constitutes a repeating unit of the SSM, as shown in Figure 8(b2). By calculating the equivalent resistivity of this repeating unit, the equivalent resistivity of the entire SSM can be obtained. Define the center-to-center distance between two adjacent diamond-shaped holes in the long axis direction as *D*_L_, the center-to-center distance between two adjacent diamond-shaped holes in the short axis direction as *D*_w_, the width of the metal wire as *D*_S_, and the thickness of the SSM as *t*_h_. Then, the length of a single current conduction path (*L*_p_) can be calculated by the following equation:

(5) $$L_{P}=2\sqrt{{\left(\frac{D_{L}}{2}\right)}^{2}+{\left(\frac{D_{W}}{2}\right)}^{2}}=\sqrt{D_{L}^{2}+D_{W}^{2}}$$

The cross-sectional area of a single current conduction path can be calculated by the following equation:

(6) $$A_{u}=D_{S}t_{h}$$

Therefore, the resistance of the repeating unit along the length direction can be calculated by the following equation:

(7) $$R_{u}=\frac{\rho_{0}}{2}\frac{L_{P}}{A_{u}}=\frac{\rho_{0}\sqrt{D_{L}^{2}+D_{W}^{2}}}{2D_{S}t_{h}}$$

where *ρ*_0_ is the electrical resistivity of 304 Stainless Steel Used in HE. An equivalent resistivity *ρ*_e_ is introduced to equate the mesh region to a macroscopic homogeneous material; therefore, the resistance of the repeating unit can also be written as

(8) $$R_{u}=\rho_{e}\frac{D_{W}}{D_{L}t_{h}}$$

Then, the equivalent resistivity can be obtained.

(9) $$\rho_{e}=\rho_{0}\frac{D_{L}\sqrt{D_{L}^{2}+D_{W}^{2}}}{2D_{W}D_{S}}$$

Since each region is composed of a number of repeating units, the equivalent resistivity of the repeating unit is equivalent to the equivalent resistivity of the entire region. The resistance of each region is then given by Equation (10):

(10) $$R_{i}=\rho_{ei}\frac{L_{i}}{W_{i}t_{h}}=\frac{\rho_{0}D_{W}L_{i}\sqrt{D_{L}^{2}+D_{W}^{2}}}{2D_{S}D_{W}W_{i}t_{h}},\;i=(1,2,3)$$

Equation (10) shows that the regional resistance is positively correlated with the short diagonal of the mesh holes and the length of the region, and it is negatively correlated with the long diagonal of the mesh holes, the width of the equivalent region, the thickness of the HE, and the width of the metal wire. These relationships provide guidance for the structural design of a perforated HE.

The total resistance *R* of the HE can be calculated after the resistance of each region is obtained.

(11) $$R=2R_{1}+\frac{R_{2}R_{3}}{2R_{3}+R_{2}}$$

Substituting Equation (10) into Equations (3) and (4), the heat generation of each region can be obtained. As region 2 and region 3 differ in size, in order to demonstrate the effect of varying mesh size on the temperature distribution within the welding area, it is necessary to compare the volume heat generation rate (*q*_Vi_) for *R*_2_ and *R*_3_, in accordance with Equation (12):

(12) $${q_{V}}_{i}=\frac{Q_{i}}{V_{i}t}=\frac{Q_{i}}{L_{i}W_{i}t_{H}t},\;i=(1,2,3)$$

where *L*_i_ and *W*_i_ are the length and width of each region.

In this study, for region 1, the *D*_L_ is 2.04 mm and *D*_W_ is 1.36 mm, L_1_ is 3.5 mm and W_1_ is 20 mm; for region 2, the *D*_L_ is 2.04 mm and *D*_W_ is 1.36 mm, L_2_ is 25 mm and W_2_ is 2.04 mm; for region 3, *D*_L_ is 3.72 mm and *D*_W_ is 2.48 mm, L_3_ is 25 mm and W_3_ is 15.92 mm. At room temperature, *ρ*_0_ is 0.72 × 10^−3^ Ω·mm. Substituting these values into Equations (3), (4) and (9)–(12), the equivalent resistivity, resistance, heat generation, and volume heat generation rate in region 2 and region 3 for the types A and B HEs can be calculated, and the results are listed in Table 2.

As shown in Table 2, the equivalent resistivities of regions 2 and 3 in the Type A HE are identical because the mesh sizes in regions 2 and 3 of the Type A HE are the same. The resistance of region 2 (660.6*ρ*_0_) is higher than that of region 3 (168.7*ρ*_0_) since region 2 is narrower. Since the resistance of region 2 is larger, more welding current will pass through region 3, making the heat generation in region 3 (73.9*I*^2^*ρ*_0_*t*) higher than that in region 2 (18.9*I*^2^*ρ*_0_*t*).

For the Type B HE, the equivalent resistivity of region 2 is 9.2*ρ*_0_, while that of region 3 is 16.8*ρ*_0_. The equivalent resistivity of region 2 is lower because the mesh in region 2 is finer, resulting in a higher proportion of metal wires and an increased number of current paths. The resistance of region 2 (362.1*ρ*_0_) is still higher than that of region 3 (168.7*ρ*_0_) because of the narrower width of region 2. The heat generation in region 2 is 21.1*I*^2^*ρ*_0_*t*, which is lower than that in region 3 (45.2*I*^2^*ρ*_0_*t*), but the heat generation per unit volume *q*v_2_ is higher than that of *q*v_3_.

A comparison of the calculation results between the Type A and Type B HEs reveals that the Type B HE significantly reduced the heat generation difference between region 2 and region 3. In the Type B HE, the heat generation in region 3 (45.2*I*^2^*ρ*_0_*t*) is twice that of region 2 (21.1*I*^2^*ρ*_0_*t*), while in the Type A HE, this difference is about four times (73.9*I*^2^*ρ*_0_*t* and 18.9*I*^2^*ρ*_0_*t*). This indicates that Type B has the ability to improve the uniformity of the temperature field, which is consistent with the temperature measurement results. Second, the heat generation in region 2 of Type B (21.1*I*^2^*ρ*_0_*t*) is higher than that in region 2 of Type A (18.9*I*^2^*ρ*_0_*t*), which is consistent with the experimental temperature measurement results, i.e., the temperature at the L-edge in the Type B was higher than that in the Type A HE. Third, as shown in Figure 6c, the resistance for the Type A HE was about 100–130 mΩ, while that for the Type B HE was about 100 mΩ. Actual measurements show that the total resistance of the Type A HE was 89.5 mΩ, with *R*_2_ of 464.3 mΩ and *R*_3_ of 157.6 mΩ. For the Type B HE, the total resistance was 62.6 mΩ, with *R*_2_ of 187.6 mΩ and *R*_3_ of 157.6 mΩ. The total resistance calculated from the voltage and welding current was slightly higher than the actual measured values because it also included the contact resistance between the HE and the copper electrodes. The resistance model calculation results show that the total resistance of Type A HE was 92.9 mΩ, with *R*_2_ of 475.3 mΩ and *R*_3_ of 121.4 mΩ. For the Type B HE, the calculated total resistance was 75.6 mΩ, with *R*_2_ of 260.7 mΩ and *R*_3_ of 121.4 mΩ. The total resistance of the Type A HE is higher than that of the Type B HE, which is consistent with the trend observed in the actual measurement results. However, there is a significant deviation (larger relative errors) between the calculated and measured resistances for the Type B HE, especially for region 2 (relative error 39.0%). We believe that this deviation is mainly due to the simplification of the resistance model in this paper. In this work, we only select the red area in Figure 8(b2) as the repeating unit. This can simplify the derivation of the model, but it also ignores many other current branches, especially for the region with a small mesh size. The region with a small mesh size will have more current branches, and a more dedicated repeating unit should be selected. For example, if Figure 8(b1) is selected as the repeating unit, it includes more current branches, making the model derivation more complex, but the results should be closer to the experiment. Despite the deviation, the overall rules predicted by the resistance model are still consistent with the experimental results in the qualitatively level, indicating that the modeling concept/idea is correct. In future research, we will further refine the resistance model to enhance its predictive accuracy. Finally, it should be noted that the resistance model cannot fully reflect the actual temperature, as it ignores the heat dissipation process caused by the resin flow and workpieces’ heat sink.

## 4. Conclusions

This study proposed a novel perforated SSM HE with tailored mesh size to mitigate the edge effect in the L-edges of the welding area on the welding interface in the resistance welding of FRTP. The joint mechanical performance and cross-sectional morphologies were investigated. The temperature evolution of the welding interface was investigated through experimental measurement and numerical calculation. A physical-based model was established to analyze the heat generation of such a tailored HE. The main conclusions can be drawn as follows.

(1) By setting a small-sized mesh outside the welding area and a large-sized mesh in the welding area, excessively high temperatures were observed near the T-edges (571 °C), and relatively low temperatures were observed near the L-edges (238 °C). Obvious weld defects can be observed near the T-edges. The average LSS of joints was 20.3 ± 1.3 MPa.

(2) By refining the mesh size near the L-edges in the welding area, the excessively high temperature near the T-edges was effectively decreased (from 571 °C to 420 °C), and the temperature near the L-edges increased (265 °C). The average LSS of joints reached 26.6 ± 0.8 MPa.

(3) The FE simulation results indicate that by reducing the mesh near the T-edge region, the current density passing through this area can be increased, thereby enhancing the heat generation in this region.

(4) The proposed resistance model shows that the regional resistance can be tailored by adjusting the mesh aperture size, metal wire width, HE thickness, open area ratio, region area, and even welding current direction (influence the welding current path). However, the proposed model can only predict the changing trends of resistance and heat generation in different regions at the qualitative level, and it still shows a relatively large deviation from the actual situation at the quantitative level, especially for the Type B HE, due to the simplification of the model.

This paper conducts a preliminary study on a novel perforated HE with tailored mesh size to mitigate the edge effect in the resistance welding of FRTP. Although the research results indicated the potential advantages of the proposed new HE, this work still has certain limitations. The demonstrated geometry–performance relationship is only established based on two types of HE and is limited to the investigated CF/PA6 welding configuration. The mesh structure and welding process require more systematic and comprehensive optimization. The geometry–performance relationship presented in this study needs to be further validated on more HE structures and welding materials. The FE model was used only for electrothermal simulation analysis of the HE, with the aim of demonstrating how adjusting the HE mesh size can alter the heat distribution of the HE. It is not used for predicting or verifying the temperature at the welding interface during the actual welding process. The demonstrated geometry–performance relationship is only established based on two types of HE and is limited to the investigated CF/PA6 welding configuration. The next work is to establish a more complete and comprehensive FE model that takes into account the flow of molten resin and the heat dissipation of the polymer films and workpieces. Although the established resistance model is consistent with the experimental results at the qualitative level, due to the simplicity of the selected repeating unit, it still shows certain errors in the quantitative calculations. In the next step, a more sophisticated repeating unit will be selected to establish a more refined resistance model, in order to achieve accurate resistance calculation and effective design of a perforated HE.

## Figures and Tables

**Table 1:** Physical parameters of 304 stainless steel.

Material	Temperature (°C)	Density (kg/m^3^)	Transversal Thermal Conductivity (W/m·K)	Electrical Conductivity (S/m)	Constant Pressure Heat Capacity (J/(kg·K))	Emissivity
304 SS	25	7858.69	15.23	1.38 × 10^6^	474.03	0.10
	100	7830.17	16.75	1.28 × 10^6^	490.89	0.12
	300	7745.87	19.44	1.09 × 10^6^	506.58	0.15
	500	7654.06	22.10	9.59 × 10^5^	529.96	0.19
	700	7558.72	24.34	8.66 × 10^5^	576.06	0.23
	900	7461.88	-	7.98 × 10^5^	619.84	0.27
	1100	7363.60	-	7.48 × 10^5^	627.54	0.31

**Table 2:** The calculated equivalent resistivity, calculated and measured resistance, and calculated heat generation in region 2 and region 3 for the type A and B HEs.

	Type A HE			Type B HE		
	Theoretical Calculation	Actual Measurement	Relative Error	Theoretical Calculation	Actual Measurement	Relative Error
*ρ*_e2_ (Ω·mm)	16.8*ρ*_0_ (1.2 × 10^−2^)	-	-	9.2*ρ*_0_ (6.6 × 10^−3^)	-	-
*R*_2_ (mΩ)	660.6*ρ*_0_ (475.3)	464.3	2.4%	362.1*ρ*_0_ (260.7)	187.6	39.0%
*Q*_2_	18.9*I*^2^*ρ*_0_t	-	-	21.1*I*^2^*ρ*_0_t	-	-
*q*_v2_	1.1*I*^2^*ρ*_0_	-	-	1.2*I*^2^*ρ*_0_	-	-
*ρ*_e3_ (Ω·mm)	16.8*ρ*_0_ (1.2 × 10^−2^)	-	-	16.8*ρ*_0_ (1.2 × 10^−2^)	-	-
*R*_3_ (mΩ)	168.7*ρ*_0_ (121.4)	157.6	23.0%	168.7*ρ*_0_ (121.4)	157.6	23.0%
*Q*_3_	73.9*I*^2^*ρ*_0_t	-		45.2*I*^2^*ρ*_0_t	-	-
*q*_v3_	1.1*I*^2^*ρ*_0_	-	-	0.6*I*^2^*ρ*_0_	-	-
*R* (mΩ)	129*ρ*_0_ (92.9)	89.5	3.8%	105*ρ*_0_ (75.6)	62.6	20.8%

## Data Availability

The original contributions presented in this study are included in the article. Further inquiries can be directed to the corresponding author.
